# Supplementary material for: The causal relationship between the human gut microbiota and pyogenic arthritis: a Mendelian randomization study
Source: Front Cell Infect Microbiol. 2024 Nov 26;14:1452480. doi: 10.3389/fcimb.2024.1452480 (PMC11629706; doi:10.3389/fcimb.2024.1452480)
Supplement: Supplementary file 1 [file DataSheet1.zip › mendelian test/ebi-a-GCST90027635.csv_leave_one_out.pdf]

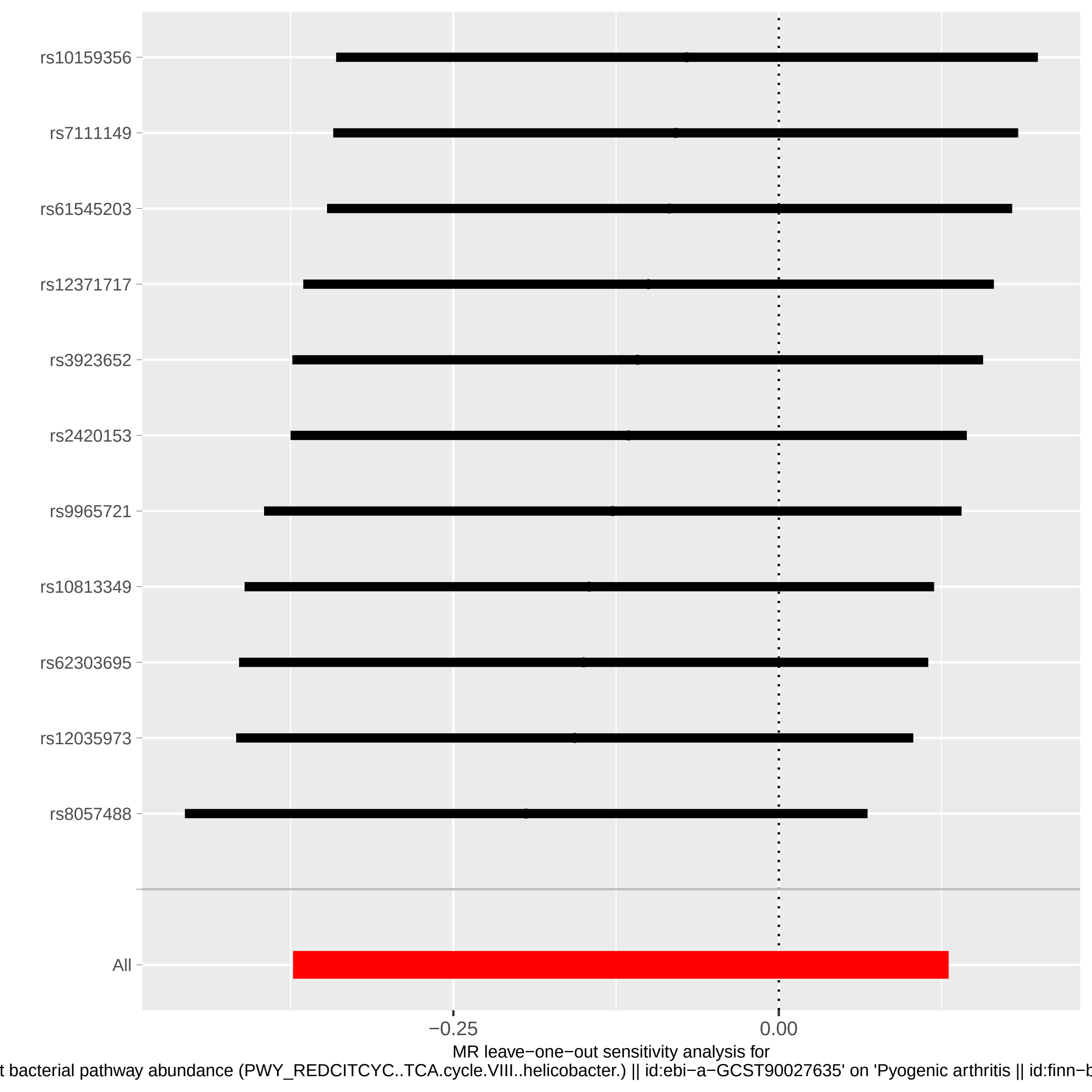

rs10159356
rs7111149
rs61545203
rs12371717
rs3923652
rs2420153
rs9965721
rs10813349
rs62303695
rs12035973
rs8057488
All
−0.25
0.00
MR leave−one−out sensitivity analysis for
t bacterial pathway abundance (PWY_REDCITCYC..TCA.cycle.VIII..helicobacter.) || id:ebi−a−GCST90027635' on 'Pyogenic arthritis || id:finn−b
